# RE: An easy, reproducible and cost-effective method for andrologists to improve the laboratory diagnosis of nonobstructive azoospermia: a novel microcentrifugation technique

**DOI:** 10.1590/S1677-5538.IBJU.2016.02.05

**Published:** 2016

**Authors:** Rosa Alice Casemiro Monteiro, Juliana Risso Pariz, Patrícia de Campos Pieri, Jorge Hallak

**Affiliations:** 1 Androscience – Pesquisa Clínica de alta Complexidade e Laboratório de Andrologia, São Paulo, Brasil; 2 Seção de Andrologia - Divisão de Urologia, Faculdade de Medicina da Universidade de São Paulo, São Paulo, Brasil; 3 Unidade de Reprodução Toxicologia - Departamento de Patologia, Faculdade de Medicina da Universidade de São Paulo, São Paulo, Brasil

*Reply by The Authors*,

*“What a piece of Work is a Man”* quoted William Shakespeare (1564–1616 AC).

The diagnosis or confirmation of azoospermia or cryptozoospermia with this nouvelle technique of microcentrifugation proposed and developed by our group is an answer to countless hours of attending difficult cases of men investigated or presenting with non-obstructive azoospermia (NOA) and having to give them a truthful perspective. It is amazing on how many false-positive or false-negative results of simplified “spermiograms” in the last 18 years we have had the difficult task to confront and disagree, unfortunately the majority of them performed in Clinical Analyses General Laboratories, so common in Brazil, not always familiarized with sperm physiology or andrology lab techniques widely disseminated by World Health Organization manuals. Surprisingly, wrongful results are not unusual from single semen analyses performed in “*andrology*” facilities located inside some Centers with eyes mainly focused in performing Intra-Cytoplasmic Sperm Injection (ICSI) as the *silver-bullet* for dealing with male infertility.

These challenges have resulted in the development of a new microcentrifugation technique carrying important elements to become a “must-do step” in the diagnosis of NOA patients - the endpoint variable of testicular failure due to, but not limited to health issues like: severe medical conditions, chronic diseases, varicocele, genetic causes, medications, illicit drugs, obesity, alcohol consumption, lifestyle habits, etc. Attention must be given to the fact that many of these conditions are potentially treatable with at least partial recovery of spermatogenesis, therefore avoiding the unnecessary use of advanced reproductive techniques such as ICSI, or using less harmful ones like simple intra-uterine insemination.

Of notice, is that an ICSI performed with a better quality sperm, after medical and/or surgical interventions by trained andrologists, will most likely result in increased success rates with less complications, such as: minor and major malformations, abortion, premature delivery or low-birth weight, enhancement of genetic disorders, hyperstimulation syndrome therefore protecting both mother and offspring and consequently, future generations. So in the era in which the myth of a “single sperm being enough” as largely and biased advertised regardless of sperm quality and functional capacity for successful fertilization and early embryonic development, is easily rebated by recent scientific data.

This efficient and simple method has the advantage of being easily performed, reproducible and cost-effective in minimum facilities andrology laboratories spread all over the World, with implications of prognostic value both for induction of spermatogenesis using medical treatments such as human chorionic gonadotropins (hCG), recombinant FSH, aromatase inhibitors, anti-estrogens such as clomiphene or more selective ones like enclomiphene, or a combination of them. Also, adds a potential positive predictive value for sperm retrieval, if that is the decision to go. Moreover, it brings implications that go far beyond a diagnostic tool, avoiding the use, for example, of donor sperm in many cases where sperm can be identified either in the initial analyses or after treatment, changing both patient's and attending physician perspectives in the initial evaluation, during treatment, in the andrology lab or in the micro-manipulation lab when “responsible ICSI” becomes mandatory.

*Jorge Hallak, MD, Ph.D* *Diretor Médico e Cientifico* *ANDROSCIENCE* *Pesquisa Clínica de alta Complexidade* *e Laboratório de Andrologia, São Paulo, Brasil* *R. Joaquim Floriano, 533 / 904* *São Paulo, SP, 04534-011, Brasil* *Telephone: +55 11 3073-0623* *E-mail: hallakj@androscience.com.br*
